# High-altitude decompression strain can be reduced by an early excursion to moderate altitude while breathing oxygen

**DOI:** 10.1007/s00421-021-04794-2

**Published:** 2021-08-19

**Authors:** Rickard Ånell, Mikael Grönkvist, Mikael Gennser, Ola Eiken

**Affiliations:** Swedish Aerospace Physiology Centre, Division of Environmental Physiology, Royal Institute of Technology, KTH, Stockholm, Sweden

**Keywords:** Altitude decompression sickness, Decompression sickness risk, Fighter aircraft, Gas bubble formation, Repeated altitude decompression, VGE

## Abstract

Recent observations suggest that development of venous gas emboli (VGE) during high-altitude flying whilst breathing hyperoxic gas will be reduced by intermittent excursions to moderate altitude. The present study aimed to investigate if an early, single excursion from high to moderate altitude can be used as an in-flight means to reduce high-altitude decompression strain. Ten healthy men were investigated whilst breathing oxygen in a hypobaric chamber under two conditions, once during a 90-min continuous exposure to a simulated cabin altitude of 24,000 ft (High; H) and once during 10 min at 24,000 ft, followed by 30 min at 15,000 ft and by 80 min at 24,000 ft (high–low–high; H–L–H). VGE scores were assessed by cardiac ultrasound, using a 6-graded scale. In H, VGE increased throughout the course of the sojourn at 24,000 ft to attain peak value [median (range)] of 3 (2–4) at min 90, just prior to descent. In H–L–H, median VGE scores were 0 throughout the trial, except for at min 10, just prior to the excursion to 15,000 ft, whence the VGE score was 1.5 (0–3). Thus, an early, single excursion from high to moderate cabin altitude holds promise as an in-flight means to reduce the risk of altitude decompression sickness during long-duration high-altitude flying in aircraft with limited cabin pressurization. Presumably, such excursion acts by facilitating the gas exchange in decompression bubbles from a predomination of nitrogen to that of oxygen.

## Introduction

Decompression strain remains a cardinal problem in several aerospace settings, for instance, during extra-vehicular space activities (“space walks”), high-altitude high-opening parachuting and, not the least, during high-altitude flying in military aircraft (McLean 2020; Stepanek and Webb 2008). In fighter aircraft, currently operating at peak altitudes ranging between 40,000 and 70,000 ft (≈ 12,200–21,300 m), the cabin-to-ambient pressure differential should not exceed 34.5 kPa (Chaurasiya et al. 2020), to prevent pulmonary overexpansion and rupture, consequent to Boyle gas expansion, in case of a sudden cabin decompression (Bjurstedt 1952; Heimbach and Sheffield 1985; Roth 1968). Thus, at these altitudes, the pilots will be exposed to ambient pressures in between 53 and 39 kPa. Therefore, and since modern fighter aircraft have in-air fueling capacity, permitting extended duration sorties at high altitude, decompression sickness (DCS) in fighter pilots is a growing concern (Westphalen 2020). We have in a series of experiments investigated the decompression strain induced by high-altitude flying (at a cabin pressure corresponding to an altitude of 24,000 ft (7315 m; cabin altitude) interrupted by excursions to cabin altitudes of 15,000–20,000 ft (4572–6096 m), which are realistic for refueling, but not sufficient to completely compress gas bubbles formed in blood and other tissues at high altitude (Ånell et al. 2019, 2020). It appears that under normoxic conditions, such excursions to moderate altitudes increase the decompression strain during subsequent exposure to 24,000 ft, presumably because the increased gas pressure during the excursions serves to replenish body tissue nitrogen (N_2_) depots and hence increase the degree of N_2_ supersaturation at high altitude (Ånell et al. 2019, 2020). During hyperoxia, by contrast, i.e. with the pilot breathing 90% oxygen (O_2_), repeated excursions to moderate altitude [18,000 ft (5486 m)] seemed to reduce the decompression strain at high altitude (24,000 ft); an effect that was attributed to facilitated gas exchange in the decompression-induced bubbles, from a predomination of N_2_ to that of O_2_ (Ånell et al. 2021). Notwithstanding, occasional incidences of DCS occurred during the 30 min at 24,000 ft, prior to the first excursion to moderate altitude (Ånell et al. 2021). In addition, it was not clear whether iterative excursions were needed to accomplish sustained reduction of the decompression strain at high altitude (Ånell et al. 2021). The aim of the present study was to further explore the possibility to use an early, single excursion from high to moderate altitude as an in-flight means to reduce the risk of altitude DCS. We hypothesised that, whilst breathing 100% O_2_, a 30-min excursion to 15,000 ft after 10 min at 24,000 ft, will reduce the decompression strain to acceptable levels during a subsequent 80-min exposure to 24,000 ft, and that the decompression strain during such time-altitude profile will be markedly lower than during a continuous 90-min exposure to 24,000 ft.

## Methods

### Subjects

Ten healthy non-smoking men volunteered as test subjects. Their mean (range) age and body mass index were 44.6 (20–54) years and 26.8 (22.0–34.1) kg/m^2^, respectively. The subjects were recruited among divers (*n* = 8) and medical students (*n* = 2). All were familiar with hypobaric chamber exposures and informed about the symptoms of DCS. One of the divers had on a previous occasion experienced DCS (type 1) in the hypobaric chamber with total remission of symptoms during the recompression phase of the exposure. The divers had an approved yearly examination for duty before the exposures and the remaining two subjects were examined by a flight surgeon prior to admission. Before giving their consent to participate, the subjects were informed that participation was voluntary and could be terminated at any given time. The experimental procedures and protocol were approved by the Swedish National Ethics Review Authority (Approval # 2021-00661).

### Equipment

All experiments were performed in a 21-m^3^ hypobaric chamber (AB Motala Verkstad. 1953, Reg. nr: 20621) at the Division of Environmental Physiology, KTH Royal Institute of Technology, Stockholm. The experiments were monitored and recorded using a video/audio system (JVC MI 5000 Victor Company, Tokyo, Japan). Each subject was breathing O_2_ from a standard 50-l bottle for compressed gas (max filling pressure: 200 bar), connected via a pressure-reduction valve to a pressure-demand full facemask regulator (Atmosphere, Poseidon Diving Systems AB, Göteborg, Sweden). To avoid high levels of O_2_ in the chamber, the expiratory side of each subject’s facemask was, via a respiratory hose (diameter 38 mm), connected to a confined space in the chamber, that was regularly ventilated throughout the exposure. In addition, the hypobaric chamber was intermittently ventilated during the course of each experiment.

VGE was detected in the right cardiac ventricle from ultrasound four-chamber cardiac images, using a phased-array transducer (1–5 MHz) and an ultrasound system (CX50, Philips Ultrasound Bothell, WA, USA). The prevalence of VGE was evaluated using the 6-graded Eftedal–Brubakk (EB) scale (0–5): 0 = no visible bubbles; 1 = occasional bubbles; 2 = at least one new bubble every fourth heartbeat; 3 = at least one new bubble every heartbeat; 4 = at least one bubble/cm^2^; 5 = “white out”, single bubbles cannot be discriminated (Eftedal et al. 2007; Nishi et al. 2003). All cardiac ultrasound videos/images were stored on a computer hard drive (Latitude E5530, Dell, USA). Cardiac Output (CO) and heart rate (HR) were measured using impedance technique (Physioflow PF07 system, Enduro, Manatec Biomedical, Paris, France) and data were stored on a computer (Latitude E5530, Dell, USA). Capillary oxyhaemoglobin saturation (SpO_2_) was monitored continuously by a pulse oximeter with the sensor placed on an index finger. (Radical 7 Monitor MASIMO SET, Rainbow CA, USA). O_2_ content in the chamber gas was monitored using an oxygen sensor (Datex Normocap 200 Oxy, Datex Finland). SpO_2_ and chamber O_2_ signals were registered using a data acquisition system (BioPac MP150, BioPac Systems Inc., USA) in combination with a computer (Latitude E5530, Dell, USA).

### Procedures

Each subject was examined on two separate occasions, interspersed by ≥ 72 h; on one occasion, the simulated altitude exposure was 90 min continuously at 24,000 ft (condition High, H) and on the other occasion, the 90-min stay at 24,000 ft was interrupted after 10 min by a 30-min excursion to 15,000 ft (high–low–high, H–L–H). The order of the two trials was alternated among subjects in a counter-balanced fashion. For the individual subject, the two trials were performed at approximately the same time of the day.

All hypobaric exposures were performed with an inside experimenter accompanying the subjects in the chamber. The subjects were instructed to avoid heavy exercise (48 h) and nicotine intake (4 h) before an exposure. Immediately prior to each experiment, the subject, who was dressed in shorts and sneakers, performed 150 unloaded squats during a 10-min period, in an attempt to equalize the amount of micronuclei in the circulation (Dervay et al. 2002). Thereafter, he was instrumented with six pre-gelled electrodes on the thorax and neck for impedance- and electro-cardiography recordings. Throughout each experiment, the subject was lying horizontally on his left side on a gurney. The subject donned the facemask upon initiation of ascent and then breathed O_2_ throughout the experiment, whereas the inside experimenter breathed O_2_ during the experiment as well as 1 h prior to ascent. Ascent and descent rates were 5000 ft/min. HR, CO, SpO_2_ and chamber O_2_ content were recorded continuously throughout each experiment, whereas the prevalence of VGE was determined intermittently, at 5000, 10,000, 15,000 and 20,000 ft during ascent and descent as well as every 5th minute at the plateau altitudes. In conjunction with all VGE determinations during ascent and descent, and also with every third determination during the altitude plateau phases, the subject performed three unloaded knee bends/extensions while in the left decubitus position. This was done to provoke release of bubbles attached to vascular endothelium into the stream of mixed venous blood (Foster and Butler 2009; Gennser et al. 2012; Jankowski et al. 2004). An exception from the 15-min interval between knee-bend provocations was the initial period at 24,000 ft in the H–L–H condition, during which a knee-bend provocation was performed after 10 min, just prior to the descent to 15,000 ft. In conjunction with each VGE determination, the subject was also asked about symptoms of DCS (discomfort/pain) and, if any, to rate their severity using a ratio scale (Borg 1982). DCS was not the primary endpoint of the experiment but any exposure was a priori set to be terminated if a subject reported DCS symptoms or the inside experimenter graded a persistent EB-score > 3.

### Statistical analyses

VGE scores are ordinal data and were converted using a rank-transformation described by Baguley (2012). The VGE scores after transformation, as well as the continuous variables (HR and CO), were analysed with a one-way analysis of variance (ANOVA). *p* < 0.05 was considered significant for all test variables.

## Results

The experiments were conducted without any DCS incidences or adverse events. In both trials, the rates of ascent and descent were kept close to the stipulated values, with changes in chamber altitude being attained within 45 s from the expected time.

### Venous gas emboli (VGE)

**Fig. 1:**
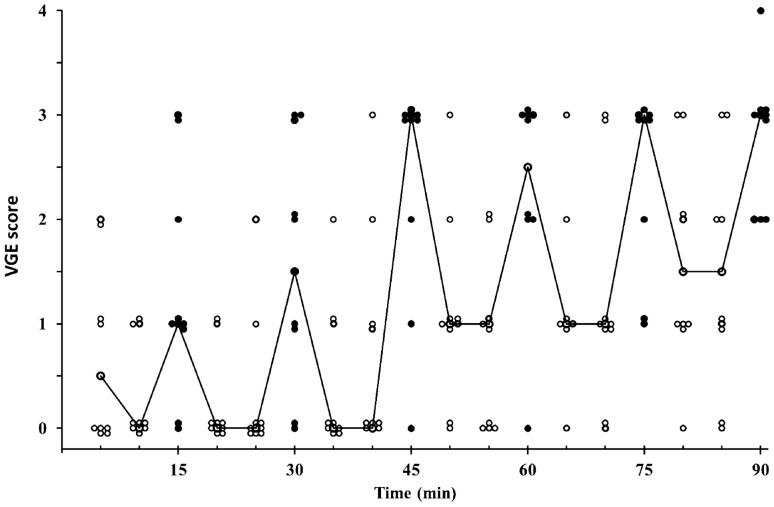
Individual and median (line) venous gas emboli (VGE) scores in Condition H (i.e. during 90 min at 24,000 ft) at rest (open circles) and following knee-bends (filled circles); *n* = 10. Thus, at each time point, a circle represents either the value of one individual (*n* = 10) or the median value (interconnected by lines)

**Fig. 2:**
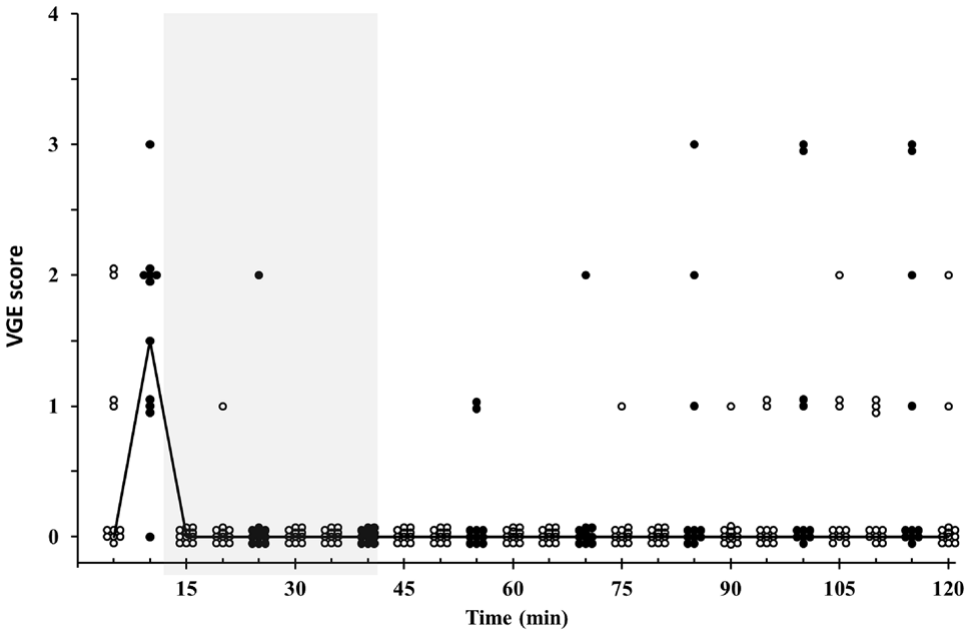
Individual and median (line) venous gas emboli (VGE) scores in Condition H–L–H, i.e. during 10 min at 24,000 ft, followed by 30 min at 15,000 ft (shaded area) and finally by 80 min at 24,000 ft. Values are at rest (open circles) and following knee-bends (filled circles); *n* = 10. Thus, at each time point, a circle represents either the value of one individual (*n* = 10) or the median value (interconnected by lines)

In the H condition, the presence of VGE increased during the course of the exposure to 24,000 ft (*p* < 0.001), with the highest values being noted at the end of the exposure, both at rest [at 85 min, median (range): 1.5 (0–3)] and after knee bends [90 min: 3 (2–4)] (Fig. 1). In the H–L–H condition, the highest VGE score was noted during knee bends after 10 min at 24,000 ft [1.5 (0–3)]. During the excursion to 15,000 ft and throughout the subsequent 80 min at 24,000 ft, median VGE scores were 0 (0–3) in the H–L–H condition (Fig. 2); there was a tendency, although not statistically significant (*p* = 0.24), of occasional bubbles reoccurring during the latter part of the 80-min post-excursion period at 24,000 ft in H–L–H (Fig. 2).

The overall prevalence of VGE during the 90 min at 24,000 ft, was markedly lower in the H–L–H than in the H condition (*F*(1,37) = 18.78, *p* < 0.001) (Figs. 1, 2). This difference was solely attributable to the last 80 min at 24,000 ft—i.e. to the post-excursion period in the H–L–H condition—with lower VGE in H–L–H than H, both at rest (*F*(1,19) = 53.3, *p* < 0.001) and following knee bends (*F*(1,19) = 32.77, *p* < 0.001) during this period (Figs. 1, 2). Whereas, during the initial period at 24,000 ft (prior to the excursion to 15,000 ft in the H–L–H condition), VGE scores were similar in H and H–L–H, both at rest and after the knee-bend provocation (which, was undertaken after 10 min at 24,000 ft in H–L–H and after 15 min in H) (Figs. 1, 2).

During the initial ascent from sea level to 24,000 ft, VGE scores were 0 at all stops (5000, 10,000, 15,000 and 20,000 ft) in both the H and H–L–H trial, whereas during the final descent, VGE was higher (*p* ≤ 0.05) in the H than in the H–L–H condition at altitude stops > 5000 ft (Table 1).

**Table 1:** Venous gas emboli scores during step-wise initial ascent from sea level to 24,000 ft and final descent from 24,000 ft to sea level, in conditions H and H–L–H

	Ascent						Descent					
	Sea level (pre)	5 kft	10 kft	15 kft	20 kft	24 kft	24 kft	20 kft	15 kft	10 kft	0.5 kft	Sea level (post)
H rest	0 (0)	0 (0)	0 (0)	0 (0)	0 (0)	0 (0)	1.5 (0–3)	1 (0–3)	0 (0–3)	0 (0–3)	0 (0–3)	0 (0–3)
H knee-bends	0 (0)	0 (0)	0 (0)	0 (0)	0 (0)	0 (0–1)	3 (0–4)	3 (0–4)	1.5 (0–4)	0.5 (0–4)	0 (0–3)	0 (0–3)
H–L–H rest	0 (0)	0 (0)	0 (0)	0 (0)	0 (0)	0 (0)	0 (0–2)	0 (0–3)	0 (0–1)	0 (0–1)	0 (0–1)	0 (0–1)
H–L–H knee-bends	0 (0)	0 (0)	0 (0)	0 (0)	0 (0)	0 (0–1)	0 (0–3)	0 (0–3)	0 (0–3)	0 (0–3)	0 (0–3)	0 (0–3)

Values are medians (range); *n* = 10

### Cardiac output (CO), heart rate (HR) and capillary oxyhaemoglobin saturation (SpO_2_)

In both conditions, CO and HR dropped substantially during the initial 15 min of the trial, and then remained relatively stable throughout the remaining course of the trial (Table 2). There was no difference in CO or HR between the H and H–L–H condition, at any given time of the trials (Table 2). In both conditions, SpO_2_ remained high (> 95%) at all times for all participants.

**Table 2:** Cardiac output (CO) and heart rate (HR) during the time at altitude in conditions H and H–L–H (24,000 or 15,000 ft). Values are means ± SD; n = 10

Variable	Time (min)	5	25	40	55	70	85	100	115
CO (l/min)	H	7.5 ± 0.2	5.7 ± 0.9	5.5 ± 0.9	5.3 ± 0.7	5.4 ± 1.3	5.4 ± 1.8	–	–
	H–L–H	7.0 ± 3.0	5.9 ± 1.7	5.5 ± 1.5	5.6 ± 1.4	5.4 ± 1.5	5.1 ± 1.4	5.1 ± 1.6	5.0 ± 1.5
HR (beats/min)	H	76 ± 8	66 ± 10	66 ± 11	64 ± 9	66 ± 10	62 ± 10	–	–
	H–L–H	76 ± 7	63 ± 9	66 ± 10	64 ± 8	63 ± 11	62 ± 11	62 ± 10	62 ± 10

## Discussion

The aim was to investigate whether, and to what degree, a single, early excursion from high to moderate altitude will decrease decompression strain, as indicated by reduced vascular bubble formation, during a subsequent prolonged exposure to high altitude. The results demonstrated that the ascents from sea level to 24,000 ft induced rapid formation of decompression bubbles, with similar presence of VGE in the H and H–L–H trials, during the initial knee-bend provocation after 10–15 min at altitude. By contrast, during the remaining 80 min at 24,000 ft, i.e. in the H–L–H condition, following a 30-min excursion to 15,000 ft, the occurrence of VGE was considerably less in the H–L–H than the H condition, in particular during the knee-bend provocations. There were no DCS incidences in any of the present experiments.

Our findings that long-term uninterrupted exposure to 24,000 ft whilst breathing a hyperoxic gas is associated with substantial and prolonged decompression strain, as indicated by the high VGE levels, is in accordance with previous reports (Ånell et al. 2021; Foster et al. 1998; Pilmanis et al. 2005; Webb et al. 1998; Webb and Pilmanis 1993). It thus appears that there is little benefit from breathing a gas mixture containing 90–100% O_2_ compared to a normoxic gas as regards the prevalence of VGE during the initial 90 min at altitudes exceeding 22,500 ft (Ånell et al. 2021; Pilmanis et al. 2005). Since decompression-dependent de novo formation of gas bubbles requires a very pronounced supersaturation of gas molecules, in blood corresponding to a pressure drop of about 110 ATA (Doolette 2009), it is generally accepted that precursors are needed to induce formation of decompression bubbles in human tissues (Doolette 2009; Vann et al. 1980; Yount 1976). Thus, the prevailing notion is that during acute exposure to altitude, VGE are formed from pre-existing gaseous micronuclei (for review, see Stepanek and Webb 2008). It must be presumed that, in the present experiments, the gas phase of such micronuclei was predominantly consisting of nitrogen (N_2_). Despite that the subjects were breathing O_2_ during the 5-min ascent and onwards, it is also likely that the initial bubble growth upon arrival at 24,000 ft was mainly due to inward diffusion of N_2_ molecules, as a result of N_2_ supersaturation in the venous blood stream and adjacent tissues. Thus, a rapid ascent from sea level to 24,000 ft whilst breathing a normoxic O_2_/N_2_ mixture, induced a whole-body washout of N_2_, from mixed venous blood via the pulmonary ventilation, that occurred at an exponentially decaying rate with a half time of about 45 min (Ånell et al. 2020). Despite a 40% higher inspired partial pressure of O_2_ in the present experiments, it is likely that N_2_ remained a salient component in the majority of the capillary beds and hence in mixed venous blood for more than 30 min at 24,000 ft. In fact, there is evidence to suggest that, even during O_2_ breathing, DCS and the growth of decompression VGE are to large extent attributable to diffusion of N_2_ molecules from tissue compartments with half-times for N_2_ washout exceeding 90 min (Conkin et al. 1987; Lundin 1960). Prolonged O_2_ breathing at 24,000 ft, will eventually, by way of diffusion, change the gas content in the decompression bubbles to O_2_, whereafter the bubbles will shrink, become unstable and disappear due to outward diffusion of O_2_ in locations where metabolism has created low PO_2_ in the bubble surroundings (Blogg et al. 2003; Hyldegaard and Madsen 1989, 2007). Judging by experiments in rodents, a noticeable reduction of bubble size following a switch from breathing air to O_2_ at altitude, may take considerable time (≫ 1 h), at least in tissues with slow washout rates of N_2_, as for instance in adipose tissue (Hyldegaard and Madsen 1989, 2007). In fact, a switch from air to O_2_ at altitude, may initially lead to growth of air bubbles injected in adipose tissue, presumably due to the greater transport capacity of O_2_ than N_2_ in blood, in combination with increasing diffusion gradient for N_2_, as the share of O_2_ molecules in the bubbles increases (Hyldegaard et al. 2001).

Even though there were no DCS incidents in the present experiments, the VGE results support the notion that a mere shift of the breathing gas from normoxic to pure O_2_ (or a hyperoxic mixture) whilst flying, is not sufficient to prevent DCS during prolonged exposure to high altitude (Ånell et al. 2021; Pilmanis et al. 2005). Pre-oxygenation protocols to wash out inert gases prior to the flight sortie is an efficient means to reduce DCS risk at high altitude (Pilmanis et al. 2005; Stepanek and Webb 2008), but is very time consuming (1–6 h) and cumbersome to undertake and hence is typically not practised by the pilots. Pre-flight whole-body vibration may be an alternative, less time-consuming means to reduce in-flight decompression strain (Elia et al. 2021); presumably such vibration preconditioning acts by reducing the amount of micronuclei precursors, although its efficacy in reducing DCS symptoms remains to be settled (Elia et al. 2021).

Our findings that in the H–L–H condition the prevalence of VGE was low throughout the altitude exposure and markedly lower in the H–L–H than H condition confirm our initial hypotheses and suggest that an alternative means to reduce decompression strain during long-duration flying at high altitude, may be an early, transient excursion to moderate cabin altitude whilst breathing a hyperoxic gas. As outlined previously (Ånell et al. 2021), we assume that the VGE-reducing mechanism of such procedure is a facilitated exchange of the gas content in the decompression bubbles from N_2_ to O_2_ domination. Thus, although the Boyle compression resulting from an altitude transition from 24,000 to 15,000 ft does not suffice to demolish decompression bubbles (Ånell et al. 2020), it will reduce their diameters by about 12%, and the associated increase in bubble surface tension and consequent 12% increase in intra-bubble pressure, as dictated by the Young–Laplace equation, will facilitate outward diffusion of N_2_ from the bubbles. In addition, the increase in total pressure, from 39 kPa at 24,000 ft to 57 kPa at 15,000 ft, during oxygen breathing will, according to Fick’s first law, accentuate the diffusion gradients for N_2_ and O_2_, by increasing the local partial pressures of these gases on the inside and outside, respectively, of the bubble membranes. Thus, the cabin-altitude excursion will accelerate the exchange of bubble content to O_2_ and hence decrease the VGE development upon the subsequent period at 24,000 ft. In addition, it cannot be ruled out that the early hyperoxic compression may have destabilized the decompression bubbles by interrupting any surface recoating towards a greater share of the bubble membranes consisting of hydrophobic proteins or other surface tension reducing molecules (Harvey et al. 1944; Swan et al. 2014).

The prevalence of VGE dropped promptly upon the excursion descent to 15,000 ft, with virtually no VGE during the knee-bend provocation after 15 min at this altitude (Fig. 2). To what extent this reflected a rapid intra-bubble gas exchange, towards an increased share of O_2_, remains to be settled. In part, compression reduction of bubble size might have contributed to the scarcity of VGE at 15,000 ft, despite a mere 12% estimated reduction of bubble radii from Boyle compression. Thus, even such relatively discrete reductions of bubble radii might render a share of the bubbles smaller than the detection limit of the ultrasound system (i.e. < 20–30 μm). That also a normoxic compression from 24,000 to 15,000 ft substantially reduced the prevalence of VGE (Ånell et al. 2021) suggests that such compression effect on VGE occurrence may not have been negligible in the present H–L–H experiments. On the other hand, that the VGE scores remained low upon re-ascent to 24,000 ft in the H–L–H condition suggests that compression alone could not explain the low VGE prevalence during the latter part of the excursion to 15,000 ft; during normoxia, re-ascent from 15,000 to 24,000 ft resulted in a prompt increase in VGE occurrence (Ånell et al. 2020). A factor that likely contributed to faster shrinkage and collapse of decompression bubbles during the present excursion to 15,000 ft is the increased “O_2_ window” induced by the elevation of ambient pressure. The synonymic terms “oxygen window” (Behnke 1967) and “inherent unsaturation” (Hills 1977) refer to the principle that the sum of alveolar and hence arterial partial pressures of gas exceeds the sum of gas tensions in the tissues, because of the oxygen metabolism in the tissues (Behnke 1967; Hills_,_ 1977). Thus, at any given level of metabolic rate, provided that alveolar PO_2_ is sufficiently high to saturate haemoglobin with O_2_ during its lung passage, which, judging by the SpO_2_ values, was always the case in the present experiments, then the oxygen window will increase as a function of increased alveolar PO_2_ (Tikuisis and Gerth 2003; Van Liew et al. 1993). Even though the effects of the O_2_ window on decompression strain are only well established for steady-state conditions (Tikuisis and Gerth 2003), which were probably not attained during the excursion to 15,000 ft, the increased O_2_ window during this period likely facilitated shrinkage of decompression bubbles. Thus, both theoretical analyses and experimental evidence suggest that an increased oxygen window will shrink decompression bubbles irrespective of the share of inert gas in the bubbles (Foster et al. 1998; Randsoe and Hyldegaard 2012; Van Liew et al. 1993).

Taken together, the present and previous studies (Ånell et al. 2019, 2020, 2021; Pilmanis et al. 2002) suggest that pressure increments induced by excursions from high (24,000–25,000 ft) to low (≤ 900 ft) altitudes will suffice to completely compress decompression bubbles, resulting in sustained (≥ 30 min) very low VGE scores during the subsequent exposure to high altitude, even if the pilot/subject is breathing normoxic gas during the excursions (Ånell et al. 2019; Pilmanis et al. 2005). By contrast, excursions from high (24,000 ft) to moderate (15,000–18,000 ft) altitude will not demolish decompression bubbles by way of compression, but will lead to sustained (> 30 min) very low VGE scores during the subsequent exposure to high altitude, if the pilot/subject is breathing a hyperoxic gas (Ånell et al. 2021), but not if he/she is breathing a normoxic gas (Ånell et al. 2019, 2020).

In the H–L–H condition, after about 40 min at 24,000 ft, following the excursion, there was a tendency (albeit not statistically significant) for partial reappearance, of VGE (Fig. 2). This may reflect the aforementioned N_2_ supersaturation in tissue compartments with slow N_2_ washout. Thus, the net effect on VGE prevalence during a longer period at 24,000 ft (> 80 min) following an excursion to 15,000 ft, with influence of the prolonged O_2_ breathing opposing that of the slow N_2_ washout compartments, remains to be established.

Even though present findings suggest that an early, single excursion/bounce to moderate cabin altitude holds promise as an efficient means to ad hoc reduce decompression stress and hence prolong a fighter pilot’s operational time at high altitude, it should be noted that the study needs to be expanded before implementing such strategy in practice. Thus, although VGE determinations are valid markers of decompression strain (Conkin et al. 1998; Eftedal et al. 2007), accounts of a causal relationship between VGE prevalence and DCS are rare (Ljubkovic et al. 2010). A substantially larger subject group needs to be investigated to establish the effect of an early excursion on the risk of developing DCS at high altitude. In addition, the protocol needs to be refined, establishing the optimal duration and altitude of the excursion as well as whether a slightly lower O_2_ content in the breathing gas, as expected in aircraft with on-board oxygen generating systems (OBOGS), would be as effective as pure O_2_.

Finally, it should be mentioned that the present technique to reduce high-altitude decompression strain by undertaking an early excursion to moderate altitude might be used not merely by fighter pilots but also in high-altitude parachute operations, during which particularly the jump-master appears to be at risk of suffering DCS (Ånell et al. 2019).
